# The impact of baseline ^18^F-FDG PET-CT on the management and outcome of patients with gastric cancer

**DOI:** 10.1259/bjr.20220437

**Published:** 2022-09-29

**Authors:** Kieran G Foley, Will Coomer, Bernadette Coles, Kevin M Bradley

**Affiliations:** 1 Division of Cancer & Genetics, School of Medicine, Cardiff University, Wales, United Kingdom; 2 National Imaging Academy of Wales (NIAW), Pencoed, United Kingdom; 3 Velindre University NHS Trust Library & Knowledge Service, Cardiff University, Wales, United Kingdom; 4 Positron Emission Tomography Imaging Centre (PETIC), Cardiff University, Cardiff, United Kingdom

## Abstract

**Objective:**

CT and staging laparoscopy are routinely used to stage patients with gastric cancer, however the role of ^18^F-fluorodeoxyglucose (FDG) positron emission tomography (PET) combined with CT (PET-CT) is uncertain. This systematic review synthesised the evidence regarding the impact of baseline PET-CT staging on treatment decisions and patient outcomes.

**Methods:**

Systematic database searches were performed without date restriction. Studies reporting data in patients with gastric adenocarcinoma who underwent radiological staging were included. One reviewer screened titles and abstracts for suitability and two reviewers extracted data from included articles. Primary outcome was the reported change in management after PET-CT. Secondary outcomes were the rates of recurrence and overall survival between patients staged with and without PET-CT. Risk of bias was assessed using the ROBINS-I tool. PROSPERO registration (CRD42022304314).

**Results:**

Data from 11 studies recruiting 2101 patients between 2012 and 2021 were included. PET-CT was performed in 1422 patients. Change of management varied between 3% and 29% of cases. No studies compared recurrence or survival rates between patients staged with or without PET-CT. Adenocarcinoma of intestinal subtype tended to be more FDG-avid compared to diffuse or signet-ring subtypes. No randomised data existed, and studies were considered low quality with high risk of bias.

**Conclusion:**

Evidence for the additional value of PET-CT in the gastric cancer staging pathway is limited. All studies reported a positive impact by preventing those with undetected metastatic disease on CT undergoing futile surgery. Future national guidelines should consider routine staging PET-CT in gastric cancer.

**Advances in knowledge:**

Studies indicated that FDG PET-CT added benefit in gastric cancer staging by detecting more distant metastases, but these studies were generally of low quality and at high risk of bias. Intestinal subtype of gastric adenocarcinoma tended to be more FDG-avid and therefore more distant metastases were subsequently detected.

## Introduction

Despite modest improvements in outcome, the overall survival of patients with gastric cancer remains poor, with 5 year survival estimated as 20%.^1^ The incidence of gastric cancer has been declining due to improved detection and treatment of *H. pylori* infections,^2^ yet many patients continue to present with metastatic disease which limits available treatment options.^3^ Patients are currently offered peri-operative chemotherapy and gastrectomy as standard first-line radical treatment of locally advanced disease.^4^

Current radiological staging pathways rely on staging CT to assess tumour and nodal staging for resectability, and to detect distant metastatic disease, which would preclude any radical treatment.^5^ CT has good accuracy for predicting resectability but is known to have suboptimal sensitivity for distant metastatic disease.^3^

Unlike oesophagogastric cancer, where positron emission tomography (PET) is routinely recommended for staging all patients deemed to be potentially curable,^6^ the role of ^18^F-fluorodeoxyglucose (FDG) PET-CT in gastric cancer is less clear. Concerns regarding the FDG-uptake of the primary tumour, sensitivity of distant metastases, cost, and resource availability have limited clinician’s confidence in this modality. As such, the Royal College of Radiologists (RCR) indications for PET-CT use in the United Kingdom (UK), which were published in 2022, lists gastric cancer as one tumour site in which PET-CT can be considered only when management may be changed by the result.^7^ Thus, a few UK centres offer PET-CT for gastric cancer staging, however the majority do not.

Staging PET-CT should be made available to all gastric cancer patients if there is evidence of improved clinical outcomes. PET-CT has the potential to reduce the number of patients that undergo futile gastrectomy, which is associated with significant co-morbidity, when distant metastatic disease is undetected on CT. However, the evidence concerning the impact of PET-CT on treatment decisions has not been systematically reviewed before.

Therefore, this review aimed to systematically search the literature and synthesise the evidence concerning the role of baseline staging PET-CT in gastric cancer staging. The primary aim was to determine how frequently PET-CT changes patient management by detecting additional disease. The secondary aims were to assess whether PET-CT changed recurrence or survival rates in patients with gastric cancer.

## Methods and materials

This study is reported according to the Preferred Reporting Items for Systematic Reviews and Meta-analysis (PRISMA)^8^ and Synthesis Without Meta-Analysis (SWiM)^9^ guidelines. This systematic review is registered with PROSPERO (CRD42022304314).

### Search strategy and selection criteria

The MEDLINE [OVID], Embase [OVID], Cochrane Library [Wiley], Scopus [Elsevier], Web of Science Core Collection [Clarivate], Cumulative Index of Nursing and Allied Health Literature (CINAHL) [Ebsco], ClinicalTrials.gov, and World Health Organisation (WHO) databases were systematically searched on 25 November 2021. The search strategy was devised using Medical Subject Headings (MeSH) and free-text terms relating to gastric cancer and PET and adapted using the rules and syntax of the other databases. The search was limited to the English language and without date restriction. No filters for study design were applied. A grey literature search was not performed. The full search strategy can be found in Appendix 1.

Inclusion criteria for studies were: (1) patients with confirmed histological cell type of adenocarcinoma or signet-ring carcinoma, (2) patients who underwent radiological staging, (3) patients who received either chemotherapy followed by surgery, surgery alone, or palliation, and (4) sufficient follow-up (at least 12 months) to determine clinical outcomes of recurrence and survival.

The Lauren classification^10^ describes intestinal *vs* diffuse and indeterminate sub-types of gastric adenocarcinoma. The World Health Organisation (WHO) classification^11^ describes papillary, tubular, and mucinous sub-types of adenocarcinomas (which are comparable to the Lauren intestinal sub-type) and signet-ring cell and other poorly cohesive carcinomas (comparable to Lauren diffuse sub-type). In addition, rarer sub-types of gastric carcinoma (*e.g.* mixed, squamous, adenosquamous) are comparable to Lauren indeterminate sub-type. Lauren diffuse and indeterminate sub-types comprise non-intestinal sub-types.^12^

Exclusion criteria were: (1) studies with a mix of primary tumour types where data pertinent to this systematic review could not be extracted, (2) patients with gastric lymphoma or other rare cell types, (3) studies investigating oesophageal or junctional tumours (Siewert Type I and II), (4) studies investigating recurrent gastric cancer, and (5) studies that used any PET tracer other than FDG.

### Data collection and extraction

The title and abstracts of studies retrieved during the search process were screened by one reviewer (KGF) for relevance to this systematic review. Full-text articles were retrieved after screening to check against the eligibility criteria. Studies that met the eligibility criteria were included. Data were independently extracted by two reviewers (KGF, WC) from the included articles and inputted into a spreadsheet designed for this review. In cases of disagreement, a consensus was reached after consulting a third reviewer. The data variables that were collected are listed in Appendix 2.

### Outcomes

The primary outcome was the effect that PET-CT had on changing initial treatment decision plans. Secondary outcomes were the rates of recurrence and overall survival between patients staged with or without PET-CT.

### Risk of bias assessment

Methodological quality of included studies was assessed with the Risk Of Bias In Non-randomized Studies—of Interventions (ROBINS-I) tool.^13^

### Statistical analysis

Data from individual studies concerning primary and secondary outcomes were synthesised using summary statistics and confidence intervals, where available, across the cohorts of patients with gastric cancer. Meta-analysis was not performed because few high-quality studies existed. Standardised metrics were used to synthesise the data and are presented in tabular format. The standardised metric for the primary outcome, was the percentage of cases in which PET-CT was perceived to change the patient management plan from that originally intended. Heterogeneity between studies and limitations in quality of evidence were highlighted in tables. Pre-specified subgroup analysis compared the proportion of cases in which distant metastatic disease was found on PET-CT. The approach described by Murad et al^14^ to the Grading of Recommendations Assessment, Development and Evaluation (GRADE) framework^15^ was used to rate the certainty in evidence for each outcome.

## Results

The systematic search discovered 4637 records, of which 2084 were duplicates. The titles and abstracts of 2553 studies were screened for inclusion. After screening, 2506 studies were excluded leaving 47 full text articles for review.

**Figure 1.**
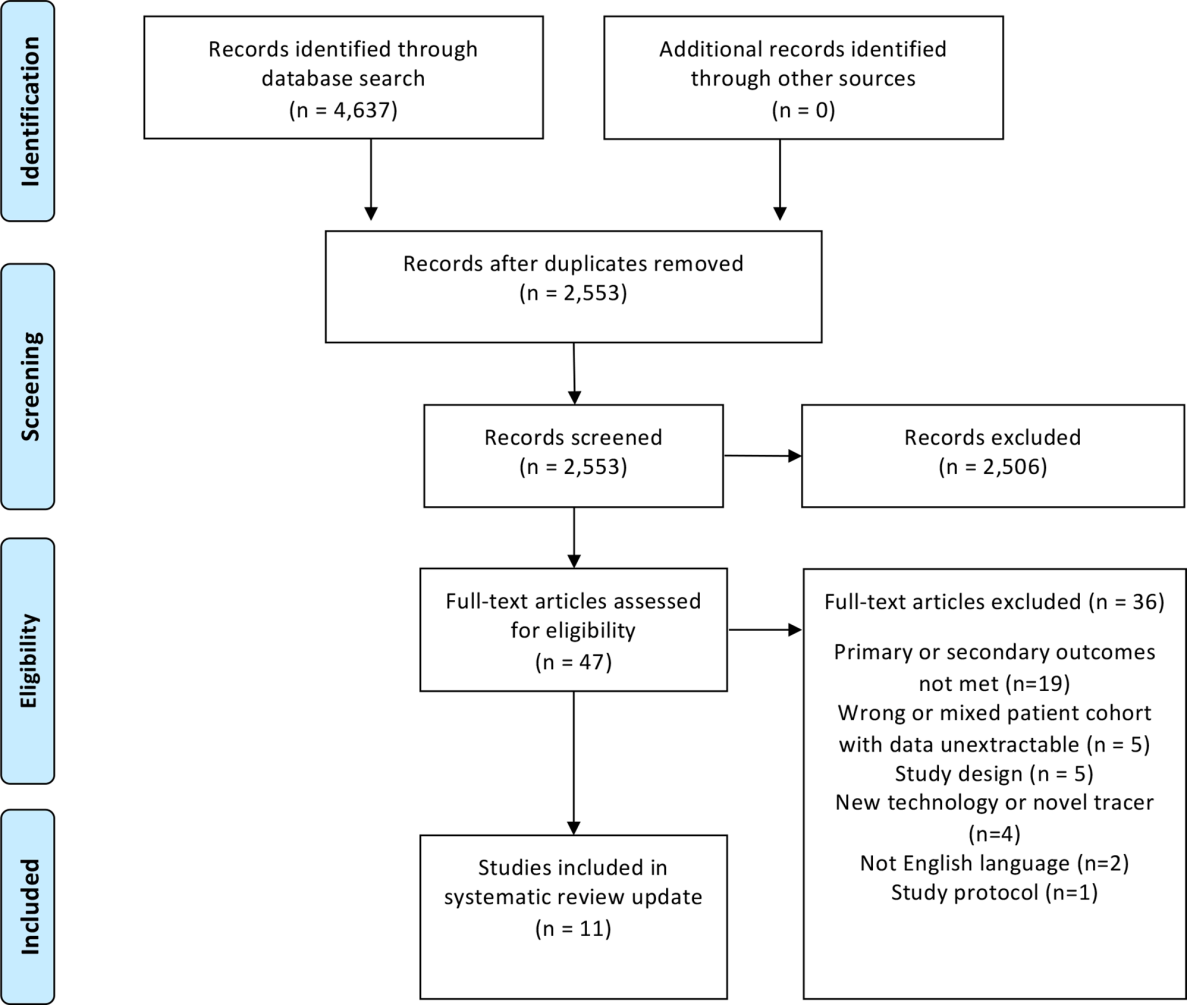
Study selection process.

19 studies did not report data relevant to the primary outcome of this review,^16–34^ 5 reported data in either patient cohorts not relevant to this review or from cohorts with mixed tumour types that could not be extracted,^35–39^ 5 had study designs that were not relevant,^40–44^ 4 investigated a new technology or novel tracers,^45–48^ 2 were not in the English language,^49,50^ and 1 was a study protocol^51^ (Figure 1).

Overall, 11 observational cohort studies^52–62^ were included that reported data from 2101 patients, of whom 1422 had PET-CT. No randomised studies were identified, and no studies reported prospective data with formal documentation of change in individual treatment decisions before and after PET-CT. Further, no studies reported differences in recurrence or survival rates in patients who received PET-CT or not. Table 1 details the important characteristics of the included studies.

**Table 1.** Summary of included cohort studies

Author	Year	Country	Study design	Sites	Recruitment dates	Cohort size	Age	Male	Number had PET-CT	Outcomes
Atici et al^52^	2016	Turkey	Prospective	1	2008–2010	31	Median (range) 57 (38–74)	23 (74.2)	31	Change in management
Bosch et al^53^	2020	England	Retrospective	1	2014–2016	330	Median (range) 73 (24–95)	214 (64.8)	105	Change in management
Debiec et al^54^	2021	Poland	Retrospective	1	2008–2019	111	Median (range) 64 (37–85)	79 (71.2)	111	Change in management
Duman et al^55^	2013	Turkey	Prospective	1	2005–2007	30	Median (range) 61 (35–80)	22 (73.3)	30	Change in management
Filik et al^56^	2015	Turkey	Retrospective	1	2011–2012	31	Mean (SD) 58.9 (12.6)	24 (77.4)	31	Change in management
Findlay et al^57^	2019	England	Retrospective	1	2007–2016	279	Median (IQR) 71 (62.3–78.0)	185 (66.3)	279	Change in management
Gertsen et al^58^	2021	Netherlands	Prospective	18	2017–2020	394	Mean (SD) 67.6 (10.7)	256 (65.0)	382	Change in management
Li et al^59^	2012	China	Retrospective	1	2007–2011	124	NR	92 (74.2)	124	Change in management
Perlaza et al^60^	2018	Spain	Prospective	1	2010–2011	50	Mean (SD) 65.7 (12.1)	30 (60.0)	50	Change in management
Serrano et al^61^	2016	USA	Retrospective	1	2006–2014	608	Median (range) 67 (29–94)	92 (55.4)	166	Change in management
Smyth et al^62^	2012	USA	Prospective	1	2003–2010	113	Median (range) 61 (25–83)	68 (60.2)	113	Change in management

IQR, interquartile range; NA, not reported; SD, standard deviation.

Most patients were male (*n* = 1,085, 65.4%). The median ages of the cohorts ranged between 57 and 71 years. Proximal tumours accounted for 249 (11.9%) of gastric cancer patients with the remainder located in the body, antrum, or pylorus. Findlay et al^57^ reported that 5.0% of patients had linitis plastica, but no other studies commented on the incidence of this pathology. Table 2 details characteristics of the PET-CT examination and scanner type in each study.

**Table 2.** Characteristics of the PET-CT examination in each study

Author	PET-CT scanner type	Dose	Length of fasting before injection (mins)	Interval between injection and scan (min)
Atici et al^52^	Siemens Biograph mCT 64	296–703 MBq	360	60
Bosch et al^53^	GE Discovery 710	350 MBq (283–389)	NR	90
Debiec et al^54^	Siemens Biograph mCT Philips Gemini XL	185–555 MBq	360	60 ± 10
Duman et al^55^	Siemens ECAT EXACT	370–555 MBq	480	60
Filik et al^56^	GE Discovery ST	8–10 mCi	360	60
Findlay et al^57^	Pre 2009: GE Discovery STE 16-slice After 2009: GE Discovery 690 64-slice From 2014: GE Discovery 710	Before 2009: 400 MBq After 2009: 4 MBq/kg	NR	Before 2009: 60 After 2009: 90
Gertsen et al^58^	Varied (no detail of individual scanners)	NR	240–360	60
Li et al^59^	GE discovery LS	10 mCi adjusted for weight	240	45–60
Perlaza et al^60^	Siemens Biograph mCT 64S	4 MBq/Kg	NR	120
Serrano et al^61^	Philips Gemini TF	5.18 MBq/Kg	>240	60
Smyth et al^62^	Siemens Biograph or GE Discovery LS	NR	>360	60

MBq, megabecquerel; NR, not reported; PET, positron emission tomography;mCi, millicurie.

All patients received ^18^F-fludeoxyglucose (FDG).

Five studies^52,54–56,59^ reported a sub-group of patients with signet ring morphology, ranging from 10 to 32% of their cohorts. Six studies reported the Lauren classification.^53,57,58,60–62^ Of the 1249 patients reported, there were similar distributions between intestinal (*n* = 578, 46.5%) and diffuse (*n* = 581, 46.5%) Lauren sub-types. Grade of differentiation was classified in 1645 patients. Most adenocarcinomas were poorly differentiated (*n* = 1052, 64.0%).

PET-CT was reported to change management in between 3% and 29% of cases. The largest cohort study,^58^ and the only multi-centre study included, reported the lowest proportion of cases (3%) where PET-CT changed the management plan (Table 3).

**Table 3.** Studies reporting the primary outcome of change in management by PET-CT

Author	Recruitment	≥T3 (%)	≥N1 (%)	PET M1	Change of management	Proportion of FDG-avid tumours	How treatment changed
Atici et al^52^	Prospective	18/31 (58.1)	12 (38.7%)	9 (29.0%)	9/31 (29.0%)	29 (93.5%)	Two from medical treatment to surgery; seven from surgery to medical treatment
Bosch et al^53^	Retrospective	NR	NR	27 (26%)	20/105 (19%)	90 (85.7%)	Became palliative with M1 disease. Upstaged N-stage in three and M-stage in 17
Debiec et al^54^	Retrospective	82 (74%)	79 (71%)	51 (46%)	13/111 (11.7%)	99 (89.2%)	Less aggressive treatment was used as result of PET-CT
Duman et al^55^	Prospective	8 (26%) *T4 only	17 (56.7%)	4 (13.3%)	2/30 (6.7%)	27 (90%)	Changed stage and surgical procedure
Filik et al^56^	Retrospective	19/31 (61.3)	13 (41.9%)	6 (19.4%)	4/31 (12.9%)	26 (87%)	6 patients had distant FDG uptake. 4 of 6 were not operated on
Findlay et al^57^	Retrospective	219/279 (78.5) *T2-4	136 (48.7%)	40 (14.3%)	20/279 (7.2%)	225 (80%)	26/279 (9.3%) of PET metastases were unsuspected on CT. Of those 26, they were unequivocal in 12 (4.3%) and indeterminate in 14. 20 PET metastases unsuspected on CT confirmed in total.
Gertsen et al^58^	Prospective	357/394 (90.6)	211 (53.6)	16 (6.8%)	12/394 (3%)	302 (79%)	Curative to palliative
Li et al^59^	Retrospective	77/124 (62.1)	84 (67.7%)	47 (37.9%)	31/124 (27.4%)	112 (90.3%)	Surgery to CRT or palliation in 34/124 (27.4%)
Perlaza et al^60^	Prospective	35/50 (70.0)	16 (32.0)	15 (30.0)	12/50 (24%)	44 (88.0%)	To avoid laparoscopy and/or futile surgical treatment. Patients had chemoradiation.
Serrano et al^61^	Retrospective	52.4% (Stage III or more)	NR	NR	31/166 (18.9%)	125 (75.8%)	PET upstaged in 31 (18.9%) and downstaged in 17 (10.3%)
Smyth et al^62^	Prospective	112 (99%)	70 (62%)	NR	11/113 (9.7%)	76 (67.3%)	Spared futile surgery

FDG, fludeoxyglucose; *M*, M-stage; *N*, N-stage; *NR*, not reported; PET, positron emission tomography;*T*, T-stage.

### Comparison of PET-CT with laparoscopy

Six studies^54,56–58,60,62^ reported the findings of PET-CT and staging laparoscopy in their cohorts. Debiec et al,^54^ Filik et al^56^ and Perlaza et al^60^ found that nine (8.1%), five (16.1%), and nine (18.0%) patients had peritoneal disease undetected by PET-CT, respectively. Gertsen et al^58^ found that 73 patients (19%) had a positive staging laparoscopy whereas only 3 patients (0.8%) in the whole cohort had FDG-avid peritoneal metastases on PET-CT. Findlay et al^57^ found that peritoneal disease was undetected by PET-CT in 35 patients (a false-negative rate of 12.5% and confirmed at laparoscopy), but in 20 patients with PET-positive metastases, 13 patients had peritoneal disease that would not have been identified at laparoscopy. No peritoneal metastases were identified by PET-CT in Smyth et al.^62^

### FDG-uptake in primary tumours

All studies reported the number of cases in which the primary tumour was deemed FDG-avid. Bosch et al^53^ found that 93% of intestinal type tumours were PET-positive *vs* 78% that were non-intestinal types, with a corresponding difference in SUVmax (14.1 ± 1.3 *vs* 9.0 ± 0.9, mean ± standard error of mean, *p* = 0.005). Similar findings were reported by Findlay et al^57^ where the median SUVmax of intestinal tumours was 8.9 (IQR 5.05–15.4) compared with 5.1 (IQR 2.50–8.10) for diffuse type tumours (*p* < 0.001). A large difference in avidity between intestinal and diffuse sub-type tumours was found by Smyth et al^62^ (97% *vs*  44%, *p* < 0.0001). Moreover, gastric adenocarcinomas with signet-ring morphology tended to have lower FDG uptake. Significantly lower FDG-uptake was found between signet-ring morphology *vs* papillary (*p* = 0.001) and tubular (*p* = 0.0008) adenocarcinoma subtypes.^60^ In Filik et al, the mean SUVmax of adenocarcinomas *vs* signet-ring tumours was 15.16 (3.00–44.60) *vs* 9.90 (5.50–17.70).^56^

### Metastatic status associated with Lauren classification

Six studies^53,54,57,60–62^ reported the distribution of metastases depending on the Lauren Classification. Findlay et al^57^ found in multi-variable analysis that after consideration of laparoscopy, patients with diffuse sub-type were less likely to have metastases overall (OR 0.86 (95% CI 0.35–2.08)), indicating greater incidence of PET-detected metastases with intestinal sub-type, although this was not statistically significant. Similarly, Bosch et al^53^ found that in FDG-avid primary tumours, the intestinal sub-type was more likely to have metastases than non-intestinal sub-types (34% *vs*  20%). Also, primary tumours with higher SUVmax (14.6 *vs* 11.2, M1 *vs* M0 stage, *p* = 0.052) were more likely to metastasise.^53^ Further, Bosch et al^53^ commented that lymph node status prediction was more accurate in intestinal than non-intestinal tumours, though overall sensitivity and specificity was poor (40 and 73%, respectively). Debiec et al^54^ reported that the risk of false-negative PET-CT for distant metastases was significantly higher for diffuse (37.5%) and signet ring (28.6%) sub-types than for intestinal sub-types (7.5%; X^2^ = 8.86, *p* = 0.003). Smyth et al^62^ found that peritoneal disease was more likely to be detected by laparoscopy than PET-CT in diffuse gastric cancer (87.5% *vs*  12.5%, *p* = 0.004).

### Risk of bias assessment

All studies were deemed at high risk of bias after evaluating each of the seven domains within the ROBINS-I tool. Further detail regarding the risk of bias assessments is included in Appendix 3.

### GRADE assessment

The certainty in evidence for the primary outcome was rated as low quality for each study according to the GRADE framework (Appendix 4).

## Discussion

Disease recurrence in patients with gastric cancer is common following gastrectomy. More than 30% of patients have recurrence within 3 years of curative resection, and the majority (88%) have distant metastases,^63^ which impacts on overall survival and quality of life. Distant metastatic disease precludes curative treatment in current UK practice. National data have reported a benefit of hepatectomy for gastric cancer liver metastases in retrospective analyses for 1 year (35.9% *vs* 50.0 %, *p* = 0.049) and 5 year mortality (61.5% *vs* 75.7 %, *p* = 0.031) *vs* no hepatectomy.^64^ However, radical treatment of oligometastatic disease is not performed routinely in clinical practice at present. Selection for gastrectomy must improve to optimise patient outcome and experience in a group of patients who historically have poor survival.^1^

This systematic review has comprehensively summarised the evidence regarding the impact that PET-CT has on patient management in eligible studies. The proportion of cases in which PET-CT changed management varied considerably across studies, ranging from 3%^58^ to 29%.^52^ Overall, the proportion of cases where PET-CT was reported to change management tended to be lower in studies with larger cohort sizes, although heterogeneity of data was noted across the included studies.

Despite all studies being graded as low-quality based on methodological limitations and risk of bias assessment, the Dutch PLASTIC study was the only prospective and multi-centre study.^58^ The PLASTIC study investigated the additional value of PET-CT and staging laparoscopy in patients with potentially curable locally advanced gastric cancer and change of management was the primary outcome. PLASTIC recruited 394 patients, of which 384 had PET-CT, and 357 had staging laparoscopy, and reported that 3% and 19% of patients had distant metastatic disease detected by PET-CT and laparoscopy, respectively.

Overall, the specificity of PET-CT for distant metastases is good, with reported values ranging between 86.5%^54^ and 100%^60^ in the studies included in this systematic review. However, the sensitivity of PET-CT varied considerably between studies from as low as 25%^52^–76.5%.^54^ A recurring limitation of the included studies was a lack of robust pathological reference standard in all patients. This is a limitation of many diagnostic test accuracy studies investigating distant metastatic disease. It would be unethical to subject all patients to an invasive biopsy with potential risk of harm to confirm a pathological diagnosis of metastasis that was highly suspected on imaging. Absence of abnormality on imaging will contribute to the true- and false-negative imaging rates, which in turn will affect the estimates of sensitivity and specificity.

No randomised data were available for PET-CT in this patient group, and generally, study quality was hindered by a high risk of selection bias, lack of pathological validation, limited clinical follow-up, and absence of documented management change. The evidence for PET-CT use in patients with gastric cancer remains limited.

Routine use of staging PET-CT in gastric cancer may avoid subsequent laparoscopy in a small number of patients and could assist the detection of metastases in difficult locations to assess during laparoscopy. Smyth et al^62^ hypothesised that if PET-CT were performed first, 11 of 113 (10%) of patients would have avoided a futile laparoscopy. Estimates of the incidence of PET-positive peritoneal disease were generally poorly reported. The main study to describe the benefits of PET-CT *vs* the limitations of laparoscopy was Findlay et al,^57^ in which PET identified unsuspected metastases in 20 patients (7.2%), in locations notoriously difficult to assess at laparoscopy, such as the lesser sac, and extra-abdominal sites.

Staging laparoscopy is not without limitations. The sensitivity and specificity of detecting macroscopic peritoneal metastases was 82% (95% CI 70–91%) and 78% (95% CI, 73–83%) in the PLASTIC study.^58^ Overall, it remains highly likely that clinicians will elect to continue routine use of staging laparoscopy in locally advanced disease, even after PET-CT, as several studies highlighted the high false-negative rate of PET-CT for peritoneal metastases. All six observational studies reported cases of peritoneal cases not detected by PET-CT.^54,56–58,60,62^ Staging laparoscopy will continue to be beneficial, particularly in the diffuse sub-type, because metastases too small to be resolved by PET are common in anatomical locations that also often abut FDG-avid structures such as liver or bowel.

There was considerable heterogeneity in the overall proportions of primary tumours that were FDG-avid ranging from 67.3%^62^ to 93.5%.^52^ Differences in FDG uptake between sub-types of adenocarcinomas have implications for the detection of distant metastatic disease. Results from included studies indicated that intestinal type gastric adenocarcinomas tend to demonstrate higher FDG uptake than non-intestinal subtypes, are more likely to metastasise, and that those metastases are more likely to be detected on PET-CT. Smyth et al^62^ reported that metastatic disease in patients with diffuse gastric cancer was more likely to be detected by laparoscopy (87.5%) than PET-CT (12.5%).

No studies reported the differences in recurrence or survival rates between patients who had staging PET-CT *vs* those who did not. Whilst a staging investigation is unlikely to directly influence these important outcomes, PET-CT may improve patient selection for radical treatment, and therefore improve overall outcomes. For instance, Findlay et al^57^ found that PET refuted metastases suggested on CT in five patients (1.79%). The clinical effectiveness of staging PET-CT in gastric cancer therefore remains unknown.

The PLASTIC study reported the numbers of incidental findings found by staging PET-CT.^58^ Of 382 patients in total, a clinically relevant lesion was found in 83 (22%) of the 132 patients with suspected relevant secondary findings, which resulted in additional investigations in 60 patients. A second primary tumour was confirmed in 7 of these 83 patients, although follow-up was not reported in the majority. Detection of incidental findings can introduce further delays into the staging pathway, which is important to be aware of. The PLASTIC study found that the introduction of staging PET-CT increased the length of staging pathway from 17 to 19 days.^58^

Few health economic data were reported in the included studies. Of direct relevance to the NHS, Findlay et al^57^ estimated that the net additional cost of PET-CT was £322.01 per patient and cost £6910.86 to avoid one futile attempt at radical treatment. Although only one, single-centre study, this suggests that PET-CT could be a cost-effective staging investigation. Data from an American healthcare system were reported in Smyth et al^62^ who concluded that if PET-CT was performed prior to laparoscopy, an additional saving of $2000 per patient could be made by reducing the need for laparoscopy in those with biopsy-proven metastatic disease.

This systematic review has synthesised the literature describing the impact of staging PET-CT on the management of patients with gastric cancer. An extensive literature search using robust methodology ensured all relevant studies reporting data pertinent to the aims of the review were captured. However, this systematic review also has some limitations. One reviewer screened the titles and abstracts of identified records for suitability. Meta-analysis was not performed because of the low-quality studies and heterogenous distribution of proportions reported within them. Study design and methodology was generally deemed to be low quality. Publication bias was not assessed but all studies reported a positive effect of PET-CT on change in management. Most studies were single-centre, retrospective in nature, and lacked sufficient detail documenting the treatment decisions made before and after PET-CT. In addition, some PET-CT scanners used during the study periods were non-time-of-flight, which results in less sensitivity and resolution of current PET scanners. Advances in digital PET technology, improved reconstruction, motion correction and the use of artificial intelligence techniques are likely to increase the diagnostic yield of small foci of disease, such as those commonly found in lymph nodes, the peritoneum, and liver.^65,66^ Therefore, the benefits of PET-CT will increase as smaller metastases are accurately detected. Future studies should aim to prospectively document treatment decisions before and after the intervention of PET-CT. Prospective studies, ideally with randomisation, should compare patient outcomes between groups who receive and do not receive PET-CT to estimate its true clinical effectiveness.

In conclusion, this systematic review has shown that evidence concerning the effectiveness of baseline PET-CT in the staging pathway of patients with gastric cancer is lacking and has identified a gap in knowledge where future research should be targeted. Several observational studies have shown that PET-CT influences management by changing treatment decisions in differing proportions of patients. Future national guidelines should consider the routine use of PET-CT in gastric cancer staging given the importance of preventing futile radical surgery.
